# The Convergence of Nanotechnology and Biotechnology in Modern Medicine

**DOI:** 10.3390/nano15030182

**Published:** 2025-01-24

**Authors:** Rúben Fernandes

**Affiliations:** 1 FCS-UFP, Faculdade de Ciências da Saúde, Universidade Fernando Pessoa, Rua Carlos da Maia, 296, 4200-150 Porto, Portugal; ruben.fernandes@ufp.edu.pt; 2 FP-I3ID, FP-BHS, Universidade Fernando Pessoa, Praça de 9 de Abril 349, 4249-004 Porto, Portugal; 3 RISE-Health, Escola de Medicina e Ciências Biomédicas, Universidade Fernando Pessoa, Fundação Ensino e Cultura Fernando Pessoa, Avenida Fernando Pessoa, 150, 4420-096 Gondomar, Portugal

The combination of nanotechnology and biotechnology is paving the way for new medical treatments, with promising results in therapy. Advances in nanomaterials have enabled the development of new drug delivery systems, more effective biomedical devices, and regenerative strategies for tissues and organs. This Special Issue, *Advances in Nanomedicine Biotechnologies*, brings together studies that illustrate the impact of this convergence, highlighting how nanomaterials can be designed for biomedical applications, from oncology to tissue engineering.

One of the greatest challenges in modern medicine is ensuring that treatments reach the correct location in the body with maximum efficacy and minimal toxicity. Nanotechnology provides a novel approach to targeted drug transport, helping to overcome common medical challenges and overcoming physiological barriers that hinder drug absorption. In oncology, Wang et al. presented advances in the use of nanostructured systems for oral drug administration in the treatment of colorectal cancer (CRC) [1]. Their study emphasizes the need to overcome gastrointestinal barriers and improve drug bioavailability. The strategy of controlled-release systems based on nanoparticles is promising for enhancing therapeutic efficacy and reducing side effects, paving the way for less invasive and more efficient treatments. The same strategy of therapeutic optimization may be applied to infectious diseases. Radenkovs et al. explored a nanostructured colloidal system based on levan and silver nanoparticles, demonstrating antimicrobial potential against multidrug-resistant pathogens [2]. Since antibiotic resistance continues to rise, researchers are exploring alternative solutions that rely less on conventional treatments. The concept of incorporating nanoparticles into therapeutic systems is also reflected in the study by Tomic et al., who synthesized a resveratrol–selenium nanocomposite (ResSeNPs) with antioxidant and antibacterial properties [3]. This approach aims to fight oxidative stress and prevent infections associated with medical implants, a crucial application in the era of hospital-acquired resistant infections.

Nanomaterials are also playing a transformative role in tissue engineering, helping to develop more effective regenerative techniques. A major challenge in tissue regeneration is ensuring that implantable materials have good biological integration and promote proper healing. In this sense, Nahum et al. present an innovative approach to the fabrication of titanium dioxide (TiO_2_) nanotubes loaded with hydroxyapatite (HAP) [4]. These nanotubes enhance osteointegration and the biocompatibility of orthopedic and dental implants, promoting bone regeneration more effectively than conventional materials. The same bioengineering rationality is applied in the study by Bedir et al., who developed 3D-printed GelMA-KerMA patches for tympanic membrane regeneration [5]. The combination of biopolymers such as gelatin methacrylate (GelMA) and keratin methacrylate (KerMA) with 3D printing enables the creation of personalized devices for repairing tympanic membrane perforations, demonstrating how nanotechnology can be integrated into tissue engineering to address complex medical conditions. Another innovative study in this Special Issue, exploring innovative biomedical applications, is that of Esmeryan et al., which investigates the impact of hydrophobic carbon soot nanoparticles on human sperm motility [6]. This study raises fundamental questions about the impact of nanoparticles on reproductive health and suggests potential diagnostic and therapeutic applications in assisted fertilization.

A key focus of these studies is how nanotechnology and biotechnology together create customized therapeutic solutions. With the evolution of bioengineering and precision medicine, nanomedicine is expected to play a central role in creating more effective therapies, smart implants, and biomaterials adaptable to individual patient needs. From new drug delivery methods to smart biomaterials, it is now clear that nanotechnology is shaping a new era in healthcare, with more effective, less invasive, and highly adaptable treatments.

The studies featured in this Special Issue emphasize the impact of nanomaterials in medicine and their growing role in personalized treatments. As research advances, it will be essential to foster interdisciplinary collaborations, bringing together engineers, biomedical scientists, chemists, and clinicians to translate these advances into accessible and effective medical solutions.
